# Mogamulizumab Combined with Extracorporeal Photopheresis as a Novel Therapy in Erythrodermic Cutaneous T-cell Lymphoma

**DOI:** 10.3390/cancers16010141

**Published:** 2023-12-27

**Authors:** Nadia Ninosu, Susanne Melchers, Max Kappenstein, Nina Booken, Inga Hansen, Maël Blanchard, Emmanuella Guenova, Chalid Assaf, Sergij Goerdt, Jan P. Nicolay

**Affiliations:** 1 Department of Dermatology, Venereology and Allergology, University Medical Center Mannheim, University of Heidelberg, 68167 Mannheim, Germany; nadia.ninosu@umm.de (N.N.); susanne.melchers@umm.de (S.M.); sergij.goerdt@umm.de (S.G.); 2 Skin Cancer Unit, German Cancer Research Center, 69120 Heidelberg, Germany; 3 Section of Clinical and Experimental Dermatology, Medical Faculty Mannheim, University of Heidelberg, 68167 Mannheim, Germany; 4 Department of Hematology and Oncology, University Hospital of Schleswig-Holstein, Campus Lübeck, 23538 Lübeck, Germany; max.kappenstein@uksh.de; 5 Department of Dermatology and Venereology, University Skin Cancer Center Hamburg, University Medical Center Hamburg-Eppendorf, 20251 Hamburg, Germany; n.booken@uke.de (N.B.); in.hansen@uke.de (I.H.); 6 Department of Dermatology, Lausanne University Hospital (CHUV) and Faculty of Biology and Medicine, University of Lausanne, 1007 Lausanne, Switzerland; mael.blanchard@chuv.ch (M.B.); emmanuella.guenova@unil.ch (E.G.); 7 Department of Dermatology, Helios Hospital Krefeld, 47805 Krefeld, Germany; chalid.assaf@helios-gesundheit.de; 8 Institute for Molecular Medicine, Medical School Hamburg, 20457 Hamburg, Germany

**Keywords:** mogamulizumab, extracorporeal photopheresis, CTCL, mycosis fungoides, Sézary syndrome

## Abstract

**Simple Summary:**

Cutaneous T-cell lymphomas (CTCLs) constitute a group of rare lymphoproliferative malignancies primarily manifesting in the skin. This study aimed to assess a combined treatment approach utilizing mogamulizumab and extracorporeal photopheresis for a subset of patients in an advanced stage of this disease, which typically challenges therapy and carries an unfavorable prognosis. The present study aimed to understand the impact of this novel treatment combination on skin- and blood-related symptoms and its associated side effects. This retrospective study included 11 patients with Sézary syndrome (SS) or mycosis fungoides (MF). Encouragingly, three-fourths of the patients showed positive responses, with improvements in skin-related symptoms, a decrease in malignant cells in the blood, and a minimum progression-free survival of 7.2 in the skin and 7.6 months in the blood. Overall, the treatment demonstrated good tolerance. If confirmed through larger-scale studies, this combined therapy could potentially establish a new therapeutic option for patients with advanced-stage cutaneous T-cell lymphoma.

**Abstract:**

Background: Primary cutaneous T-cell lymphomas (CTCLs) are rare lymphoproliferative malignancies characterized by significant morbidity and mortality in advanced disease stages. As curative approaches apart from allogeneic stem cell transplantation are lacking, establishing new treatment options, especially combination therapies, is crucial. Methods: This retrospective study included 11 patients with SS or MF receiving therapy with mogamulizumab in combination with ECP from four European expert centers. The response rates in the skin and blood as well as treatment use and adverse events (AE) were described. Results: 8/11 patients (73%) showed an overall response (OR) in the skin. The mean mSWAT decreased from 98.2 ± 40.8 to 34.6 ± 23.8. The overall response rate (ORR) in the blood was 64% with two complete responses. During combination therapy, the mean number of Sézary cells decreased from 3365.3 × 10^6^/L before treatment to 1268.6 × 10^6^/L. The mean minimum known period without progress was 7.2 months in the skin and 7.6 months in the blood. The most common AEs were mogamulizumab-associated rash (MAR) (45.5%), anemia (27.3%), lymphocytopenia (27.8%), and infusion related reaction (16.7%). No AE led to treatment discontinuation. Conclusions: Our study presents the combination of mogamulizumab and ECP as an effective therapy in the blood and skin in CTCL with good tolerability, similar to mogamulizumab monotherapy.

## 1. Introduction

Primary cutaneous T-cell lymphomas (CTCLs) are rare malignancies of the non-Hodgkin lymphoma class with an estimated incidence of 1/100,000 inhabitants per year [1,2,3]. The most prevalent form of CTCL is mycosis fungoides (MF), accounting for 50 to 70% of all cases. It typically manifests as a localized skin condition, characterized by patches, plaques, and tumors, along with symptoms such as scaling and intense pruritus [1,2,4].

Sézary syndrome (SS) represents an erythrodermic variant of CTCL with hematological involvement. It is a particularly aggressive form of CTCL, characterized by the presence of atypical malignant Sézary cells in the blood, lymph nodes, and skin. Although rare and accounting for only about 5% of all CTCL cases, it is associated with the most severe symptoms and a poor prognosis [5,6]. Overall survival for patients with SS is typically between 48 and 63 months, and the 5-year survival rate can be as low as 28% [7].

The choice of therapy depends on the stage of the disease and the latest evidence-based guidelines [8]. Mogamulizumab, a monoclonal antibody directed against C-C-chemokine receptor 4 (CCR4) expressed on malignant and regulatory T-cells in the skin and peripheral blood, is approved for the treatment of MF and SS [9,10]. Mogamulizumab is most effective against the malignant cell population in the blood, resulting in the highest response rates in patients with SS.

The phase III MAVORIC study [9] leading to the approval of mogamulizumab was an open-label, multicentric, randomized trial employing a parallel-group design (1:1) to evaluate the effectiveness and safety of mogamulizumab in previously treated adults with MF or SS compared to vorinostat, a histone deacetylase inhibitor (HDAC inhibitor). Notably, the study demonstrated a 68% partial response in the blood following mogamulizumab treatment. Recently, in September 2023, real-world data from France were published [11], which reported even more promising outcomes, showing an overall response rate (ORR) in the blood of 81.8% through mogamulizumab monotherapy.

However, despite favorable outcomes in the blood, responses on the skin manifested at comparatively lower rates, as evidenced by the MAVORIC trial, where only 44% of patients exhibited a partial response. Further corroborating this trend, the real-world data from France reported an ORR of 57% in the skin. Additionally, an important measure of treatment effectiveness, the median progression-free survival (PFS) recorded in the MAVORIC trial, stood notably at 7.7 months, emphasizing the temporal aspect of treatment efficacy.

Therefore, combination therapies including mogamulizumab and skin-directed therapies are being established, e.g., the MOGAT study combining mogamulizumab therapy with total skin electron beam therapy (TSEBT) (NCT04128072).

For stage III of MF defined by erythroderma, extracorporeal photopheresis (ECP) is a first-line treatment option. ECP is an effective and safe therapy with minimal side effects [12]. ECP is usually performed on two consecutive days every 2–4 weeks. The first study on ECP in patients with CTCL was published in 1987 by Edelson et al.; in 2012, a reanalysis of the data from this seminal study was conducted using updated international criteria, revealing a significant 74% ORR specifically concerning skin manifestations [13,14].

Information regarding the effectiveness of ECP specifically on blood is not extensive. Various studies have reported a broad range of overall response rates spanning from 42% to 80% [12,15]. However, the absence of comprehensive, large-scale investigations directly assessing its efficacy within the blood compartment poses challenges for making precise comparisons.

In addition, combining ECP with other adjunctive treatments such as interferone (IFN)-α-2a, methotrexate, bexarotene, dimethyl fumarate, or PUVA can be beneficial [12,16]. Given the poor survival rates of patients with SS and the limited treatment options available, the development of new therapies is crucial.

Additionally, Campbell et al. demonstrated that first-line combination therapies have a longer time to next treatment (TTNT) than monotherapies and that patients benefit from an early inclusion of ECP in their therapeutic algorithm [17].

Therefore, this study aims to identify a possible early combination therapy that patients derive high benefit from.

Combining ECP therapy with mogamulizumab appears to be a promising approach, since ECP has shown to be effective on the skin with response rates of around 60–70% and has a favorable side effect profile [12,18,19]. This study reports on 11 patients with CTCL who were treated with mogamulizumab in combination with ECP.

## 2. Patients and Methods

In this retrospective, observational analysis, 11 patients with erythrodermic CTCL (1 MF; 10 SS) who received mogamulizumab in combination with ECP between 2020 and 2023 at 4 different centers were included. The participating centers were the Department of Dermatology of the University Medical Center Mannheim, Germany; the Department of Dermatology of the University Medical Center Hamburg-Eppendorf, Germany; the Department of Dermatology, Helios Hospital Krefeld, Germany; and the Department of Dermatology, University Hospital Lausanne, Switzerland. This study was conducted according to ethical and GCP guidelines and approved by the Research Ethic Committees of each institution (Mannheim 2021-808).

### 2.1. Evaluations

Patient history and clinical parameters were assessed at each expert center. We centrally evaluated responses according to the consensus global response criteria for CTCL and compared them to the initial baseline assessments before combination therapy [20,21,22].

To quantitatively evaluate the burden of skin disease, we applied the modified severity-weighted assessment tool (mSWAT). The baseline mSWAT was known for 10/11 patients, but the treatment response was known for all 11 patients and thus included in the evaluations. Sézary cells were measured using flow cytometry and defined according to international guidelines as CD4+ CD7− or CD4+ CD26− cells [21,23]. The mean minimum period without known progress was calculated as mean of time to progress for patients with observed progressive disease (PD) and full observation period for patients without PD.

Adverse events (AEs) were evaluated following the guidelines of the National Cancer Institute Common Terminology Criteria for Adverse Events (CTCAE-NCI), version 5.0.

### 2.2. Statistics

The statistical analyses were performed using MS Excel and GraphPad Prism. The level of significance was set at *p* < 0.05. Student’s *t*-test or ANOVA were performed as applicable.

## 3. Results

### 3.1. Baseline Patient Characteristics

A total of 11 patients were included, thereof 10 patients with SS and 1 patient with erythrodermic MF. The mean age was 72.0 (SD 9.2) years. Of the total, 64% of patients were male. Patients had stage IIIA–IVB, of which 91% had stage IV disease. The mean disease duration was 1.8 years (SD 1.4). Five patients received mogamulizumab monotherapy before the combination treatment, and five patients directly received the combination with ECP without prior mogamulizumab monotherapy. One patient received mogamulizumab before further treatments including brentuximab, ECP and interferon, TSEBT, and then the combination of mogamulizumab and ECP.

The dose of mogamulizumab was applied according to the manufacturer’s recommendations for all patients, which is 1 mg/kg of mogamulizumab administered as an intravenous infusion over a minimum of 60 min. The administration took place weekly on days 1, 8, 15, and 22 of the first 28-day cycle. Subsequently, infusions were provided every two weeks on days 1 and 15 of each subsequent 28-day cycle. The intervals of ECP varied between every 2 weeks and every 4 weeks. Four patients received ECP every 2 weeks, five every 4 weeks, and one patient received ECP every 2 weeks for the first 2 months and then every 4 weeks.

All patients had at least 2 prior systemic therapies; 9 patients had at least 3 prior systemic therapies. All but one patient had already been treated with ECP in the past. All but 3 patients had blood involvement at the start of combination therapy. On average, patients had a mean of 12.5 (SD 11.3; median 8) sessions of combination therapy. The baseline mSWAT was 98.2 (SD 40.8). The mean Sézary cell count was 3365.3 × 10^6^/L. Further baseline characteristics are shown in Table 1 and Table 2.

The treating physicians’ reasoning behind combining treatments was hope of better response to treatment in 63.4%, progression upfront in 18.2%, severe skin involvement in 1 patient (9.1%), and reduction in pruritus and blood tumor burden in another patient (9.1%). One patient received TSEBT in addition to combination therapy, and another patient received etoposide in addition to combination therapy.

### 3.2. Clinical Efficacy

**Figure 1:**
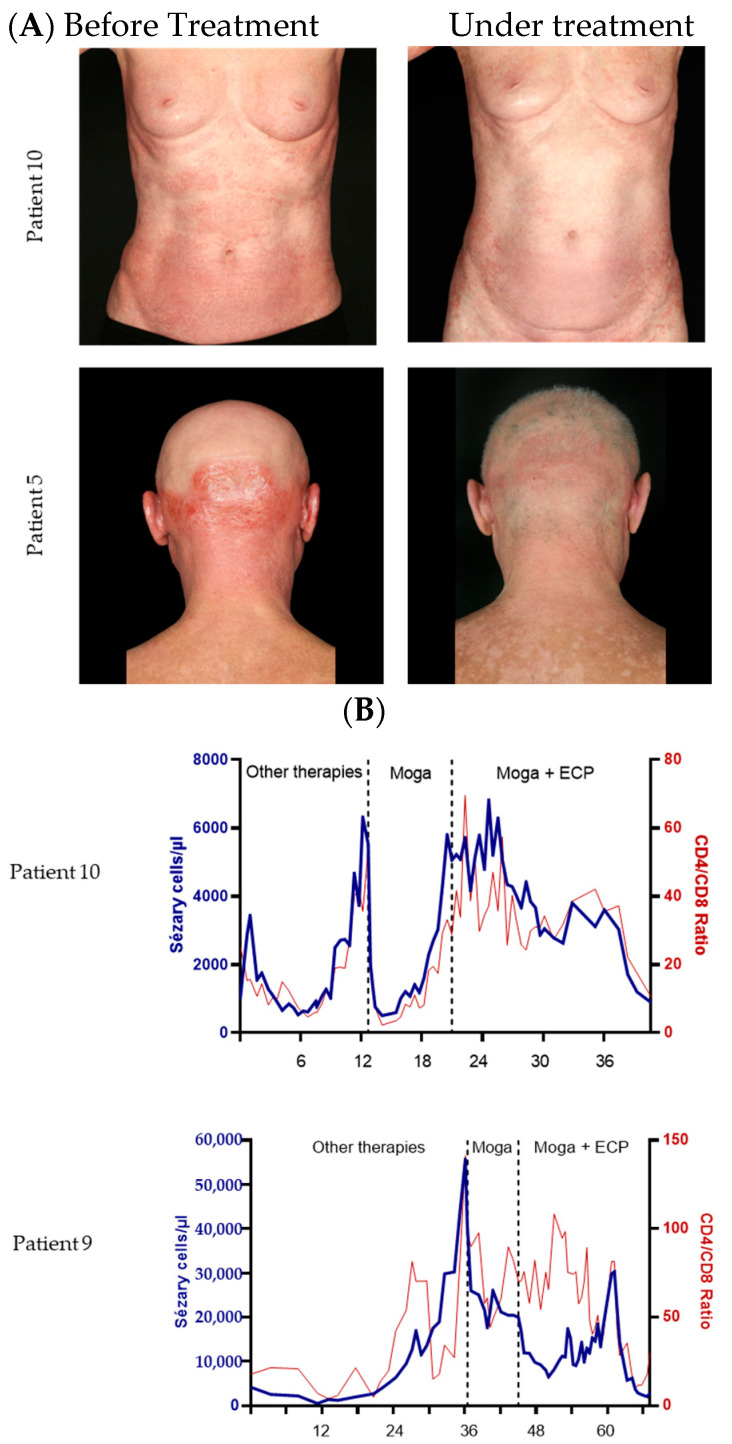
Efficacy of the combination of mogamulizumab and ECP. (**A**) Representative pictures of patients 10 and 5 before treatment and after 9 months and 1 month of combination treatment. (**B**) Representative time curves of the Sézary cell count and CD4/CD8 ratio of patient 10 and 9. Depicted are the values since the first presentation in our hospital. The treatment period is shown in months.

We mainly assessed responses in the skin and blood. Detailed results are shown in Table 3. In the skin, most of the responders showed deep responses, as depicted in the representative clinical pictures (Figure 1A).

**Figure 2:**
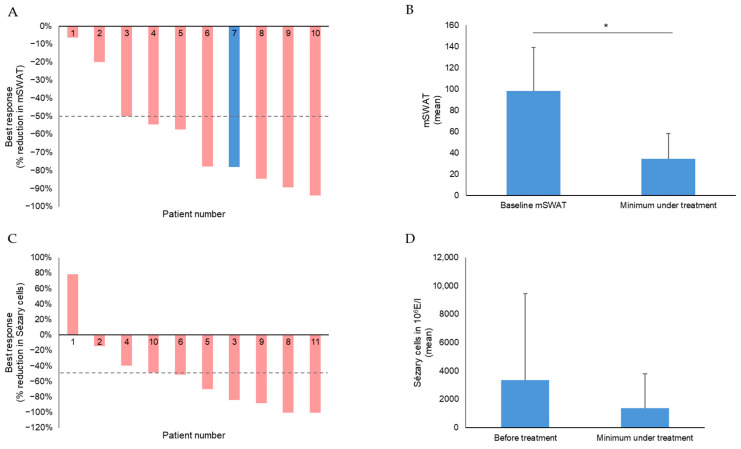
Efficacy in skin and blood. (**A**) Waterfall plot of best response in the skin, depicted by a reduction in mSWAT, in 10 of 11 patients (in 1 patient, the respective data were not available); (**B**) mean mSWAT values of 10 erythrodermic CTCL patients before and after combination therapy with mogamulizumab and ECP (in 1 patient, the respective data were not available). Differences were considered significant at *p* < 0.05 and are depicted by asterisks, with * for 0.01 < *p* ≤ 0.05. (**C**) Waterfall plot of blood response in Sézary patients reflected by reduction in Sézary cells in %. (**D**) Mean quantitative absolute Sézary cell values of 10 Sézary patients before and after combination therapy with mogamulizumab and ECP.

The mean mSWAT decreased highly significantly from 98.2 (SD 40.8) to 34.6 (SD 23.8), implying a mean decrease of 65% (absolute 63.6; SD 40.9) (Figure 2A,B). No patient showed complete response (CR) but 8/10 patients with known baseline mSWAT showed a deep partial response (PR) as best response, 1 patient stable disease (SD), and 2 patients had progressive disease (PD). Two patients had a PD during the observation period after initially responding with PR. The overall response rate (ORR) in the skin was 73%. The mean time until partial response was 3 months.

Regarding blood involvement, 2 patients showed a CR in the blood, 5 patients showed a PR, and 3 patients had SD. Additionally, one patient’s response was categorized as progressive disease (PD) in the blood, primarily due to his initial B0 status before undergoing combination treatment. Thus, the ORR in the blood was 64%. The mean time to response in the blood was 4.9 months. The mean time to response was 7.1 months for CR, 4.0 months for PR and 1.0 months for PD (Table 4). The data on lymph node and viscera involvement were not evaluated because they could not be systematically assessed in all patients due to the retrospective character of this study.

During combination therapy, the mean number of Sézary cells decreased from 3365.3 × 10^6^/L before treatment to 1268.6 × 10^6^/L after treatment (Figure 2C,D), also reflecting that in several patients, the responses in the blood were very deep, as representatively shown in Figure 1B. Here, the synergistic effect of the combination is also reflected by its additional effect on Sézary cell number compared to the mogamulizumab monotherapy before (Figure 1B).

The mean observation period was 7.8 months (range: 1.8–19.1 months; SD 6.2). The mean minimum period without known progress was 7.6 months in the blood and 7.2 months in the skin. Treatment was discontinued in 2 patients with a subsequent change to gemcitabine for both. Median overall survival or duration of response were not assessable due to the limited observation period. Time to next treatment was not calculated, as only 2 out of 11 patients received a subsequent treatment, 1 after 1.8 months, the other after 3.0 months.

### 3.3. Safety

**Figure 3:**
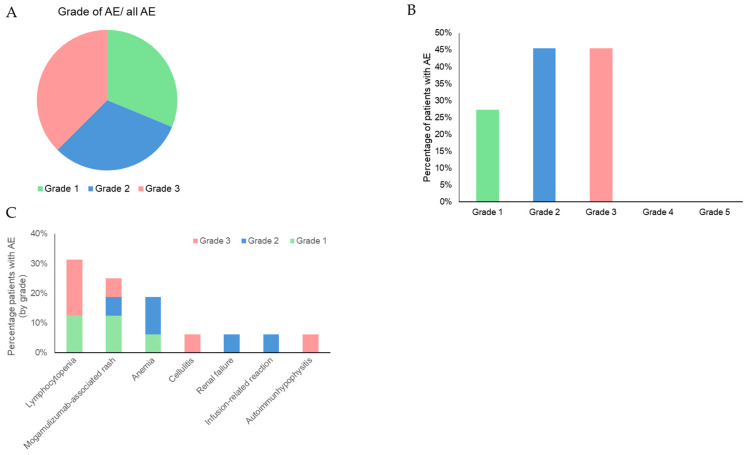
Adverse events under mogamulizumab and ECP combination therapy. (**A**) AE sorted by CTCAE grade. Depicted is the percentage of AE of each grade from total AE. (**B**) Percentage of patients who suffered from AE of each CTCAE grade. (**C**) Most frequent AE under mogamulizumab and ECP therapy sorted by frequency and depicted as the percentage of patients who suffered them including CTCAE grade.

Of the total, 90.9% patients had adverse events (AE), 33.3% of the AEs were classified as mild, 6 of all AEs were classified as severe AE (CTCAE grade ≥ 3) (Figure 3A), and 5 out of 11 patients (45.5%) experienced AE of grade 3 (Figure 3B). The AEs classified as CTCAE grade 3 were lymphocytopenia, hypophysitis, and mogamulizumab-associated rash (MAR). No patient experienced AE grade 4 or 5.

The main side effects observed were MAR (45.5%), anemia (27.3%), lymphocytopenia (27.8%), and infusion-related reaction(16.7%). All AEs with their respective CTCAE grades are shown in Figure 3C. All AEs could possibly have been induced or aggravated by the combination treatment. Due to the retrospective character of this study, it was not possible to clarify this in more detail, but all observed AEs except for autoimmunhypophysitis are described for either mogamulizumab or ECP therapy in the literature [9,11,24,25]. No AE led to treatment discontinuation.

## 4. Discussion

CTCLs present a therapeutic challenge, as they are generally considered incurable diseases apart from allogeneic stem cell transplantation. Current treatment options usually only lead to a PR, which is of limited duration and often does not last longer than one year. The time before the need for subsequent treatment (TTNT) is typically not more than 5 to 6 months [6,26,27].

Combining mogamulizumab and ECP showed promising clinical efficacy in this study with an ORR of 73% in the skin of the patients with a mean decrease of the mSWAT from 98.2 to 34.6. In comparison, the phase 3 MAVORIC study showed a partial response in 44% of patients who had received mogamulizumab and in 22% of patients who had received vorinostat [9].

Real-world data from France assessing the efficacy of mogamulizumab were recently published. An ORR was achieved in the skin of 66.7% of patients with SS and in 46% in patients with MF [11]. The first study on ECP in patients with CTCL was published in 1987 by Edelson et al.; data from this study were reanalyzed in 2012 using the current international criteria and it was found that the ORR in the skin was 74% [13,14].

Our results suggest that the combination of mogamulizumab and ECP shows superior efficacy in cutaneous involvement compared to approved targeted therapies for MF/SS, including the monotherapy of mogamulizumab and brentuximab vedotin [9,11,28]. This observation holds true even when compared to recently published phase I and phase II studies investigating innovative drug candidates such as tenalisib, dimethyl fumarate, or pembrolizumab for CTCL [16,29,30].

In the blood, the ORR was 73%, while in the MAVORIC study, the ORR in the blood was 68% with mogamulizumab treatment and 10% with vorinostat treatment. The real-world data showed a better ORR of 81.8% for monotherapy with mogamulizumab. The results in the blood of patients with the combination therapy of mogamulizumab and ECP seem to be comparable to the mogamulizumab monotherapy. Data on the efficacy of ECP on the blood are limited. Various studies show ORRs of 42–80% [12,15]. To our knowledge, the efficacy on the compartment blood itself has not been systematically investigated in larger studies, rendering comparisons difficult.

Six of eleven patients had previously received mogamulizumab treatment, and ten of eleven patients had received ECP treatments. Both preceding treatments were discontinued due to insufficient treatment response or disease progress; however, the patients responded to the combination treatment: notably, out of five patients who had been treated with mogamulizumab monotherapy shortly before the start of combination therapy, five showed a PR in the skin as best response and three patients had PR in the blood, while two patients showed a stable disease through combination treatment. We concluded that the combination treatment is effective even in patients for whom mogamulizumab and ECP monotherapy were not sufficiently effective anymore, suggesting a synergistic effect.

The course of the Sézary cells in Figure 1B also shows that the combination therapy of mogamulizumab and ECP can be an effective addition if the monotherapy with mogamulizumab was not sufficient.

The treatment of MF/SS often requires a multifaceted approach. Many patients who undergo ECP also receive additional therapies, where IFN-α-2a and bexarotene are commonly used. Research suggests that combining ECP with complementary therapies may lead to better response rates [12,31]. Also, it appears that patients benefit from early inclusion of ECP to their treatment regime [17].

As previous studies suggest, mogamulizumab is most effective against the malignant cell population in the blood, resulting in the highest response rates in patients with SS, while the response on the skin is comparatively lower. However, ECP shows favorable outcomes in terms of skin response. Our findings strongly suggest that the combination of ECP and mogamulizumab is synergistic, with strong results in the skin and blood, and therefore seems to be superior to monotherapy. This is in line with a recently published monocentric report on the combination of mogamulizumab and ECP in seven patients where the authors demonstrated effectiveness in treatment in six out of seven patients. In line with the present study, the respective responses in skin and peripheral blood underwent a parallel course in most cases [32].

A limitation of our study is the relatively brief mean observation period of 7.8 months. It is worth noting that only two patients discontinued therapy, receiving a subsequent treatment with a TTNT of 1.8 and 3 months, which is too short to evaluate the results of the combination therapy. Remarkably, the mean minimum period without known progress in the blood was 7.6 months and 7.2 months in the skin, which are very close to the mean observation period of 7.8 months. In comparison, the median duration of follow-up was 17 months in the MAVORIC study and investigator-assessed median progression-free survival was 7.7 months, while according to independent review, median progression-free survival was 6.7 months [9]. Therefore, the actual progression-free survival of the combination therapy is likely considerably longer, which should be validated in appropriate studies with sufficiently large cohorts.

Regarding the safety profile, AEs were reported in 90.9% of cases; this is higher than in the MAVORIC trial (84.4%) and real-world data (56.5%). The latter might be due to an underreporting of AEs in noninterventional studies. However, the number of patients with AE grade ≥ 3 (45.5%) was comparable to treatment with mogamulizumab monotherapy in the phase 3 study MAVORIC (41%) [9]. The main side effects observed were MAR (45.5%), anemia (27.3%), lymphocytopenia (27.8%), and infusion-related reaction (16.7%), compared to the real-life data, where MAR was described in 14.5% (SS 18.8%), lymphocytopenia in 23.4% (SS 30.4%), and infusion-related reaction 2.1% (SS 15.9%) of cases [11]. In a meta-analysis comparing 14 studies where mogamulizumab was administered for different types of cancers, the most common AEs in patients receiving mogamulizumab in combination with other drugs were neutropenia (event rate (ER) of 0.812), anemia (ER of 0.687), lymphocytopenia (ER of 0.619), and gastrointestinal disorder (ER of 0.599). Lymphocytopenia (ER of 0.568) was the most common AE of grade, ≥3 [25]. In our study, all AEs could possibly have been induced or aggravated by the combination treatment, but to our knowledge, autoimmunhypophysitis has not been described in the literature as AE related to mogamulizumab or ECP therapy. No AE led to treatment discontinuation, reflecting the favorable tolerability of the combination.

Furthermore, one of our patients received TSEBT in addition to the combination of mogamulizumab and ECP after progression in the skin, which led to a remarkable improvement in the skin. Another patient received etoposide, which was well tolerated. Both additional therapies did not change the overall response, as the best response was reached before the start of the additional therapy.

Further limitations of this study include its retrospective character and the lack of a standardized control group. However, the number of patients included was relatively small yet sizeable in light of the rarity of the disease.

Our experience shows that it is beneficial to combine mogamulizumab directly with ECP or add ECP if the response to mogamulizumab monotherapy is not sufficient; additional combination with TSEBT or etoposide was well tolerated.

## 5. Conclusions

In conclusion, our study suggests that the combination of mogamulizumab and ECP offers an effective treatment option for both blood and skin manifestations in erythrodermic CTCL with similar tolerability and a similar effect in the blood to mogamulizumab monotherapy but stronger cutaneous efficacy.

## Figures and Tables

**Table 1:** Patient characteristics.

Attribute	Attribute Level
**Sex**	**n (%)**
Female	4 (36%)
Male	7 (64%)
**Age** (mean; in years ± SD)	72.0 ± 9.2
**Ethnicity**	**n (%)**
Caucasian	11 (100%)
**ECOG**	**n (%)**
0 1 2	3 (27%) 6 (55%) 2 (18%)
**Time since initial diagnosis** (mean; years ± SD)	1.80 (±1.44)
**Disease type**	n (%)
Mycosis fungoides	1 (9%)
Sézary syndrome	10 (91%)
**TNMB stage before combination treatment**	n (%)
**T** T3 T4	1 (9%) 10 (91%)
**N** Nx N0 N3	7 (64%) 2 (18%) 2 (18%)
**M** M0	11 (100%)
**B** B0 B2	1 (9%) 10 (91%)
**CTCL stage before combination treatment** IIIA IVA1 IVA2	1 (9%) 8 (73%) 2 (18%)
**Sézary cells before combination treatment** Mean (in 10^6^ E/L ± SD)	3365.3 (± 6103.2) (n = 10)
**Lymyphocytes before combination treatment** Mean (in 10^6^ E/L ± SD)	3728.7 (± 6032.3) (n = 11)
**CD4/CD8 ratio before combination treatment** Mean (± SD)	41.2 (± 31.9) (n = 10)
**LDH before combination treatment**	
Mean (in U/L ± SD)	290.0 (± 62.1) (n = 7)
**mSWAT before combination treatment**	
Mean (± SD)	98.2 (± 40.8) (n = 10)
**Number of combination sessions** Mean (± SD)	12.5 (± 11.3) (n = 11)
**Observation period (months)** Mean (± SD) Minimum Maximum	7.8 (±6.2) 1.8 19.1

**Table 2:** Treatment history.

Therapy	First Line (*n*)	Second Line (*n*)	Third Line (*n*)	Previous Treatments (*n*)
Antibiotics	0	1	0	0
Syst. PUVA	2	0	0	1
Toctino	0	1	0	0
BXT	2	1	1	0
Brentuximab	0	1	2	2
Mogamulizumab	0	1	0	1
MTX	2	1	2	2
PDN	0	0	0	0
Chlorambucil	0	0	0	0
ECP	7	4	5	5
Dimethyl fumarate	0	0	0	2
TSEBT	0	0	0	1
Interferon	2	4	3	4

Note: The aggregated number of previous treatments can be higher than the number of patients included as the treatments were partially administered as combination treatments.

**Table 3:** Results in skin.

	Attribute	Share (%)
**Best Response** (*n*)		
CR	0	0%
PR	8	73%
SD	1	9%
PD	2	18%
**PD** (*n*) In total	4	36%
PD after initial response	2	27%
**Mean time** (*months*) to…		
CR	n/a	
PR	3.0	
PD	4.8	
**Mean minimum known period witho ut progress** (*months*)	7.2	
**Minimum mSWAT after combination treatment**		
Mean (± SD)	34.6 (±23.8)	
**Decrease in mSWAT**		
Mean (± SD)	63.6 (±40.9)	

**Table 4:** Results in blood.

	Attribute	Share (%)
**Best Response** (*n*)		
CR	2	18%
PR	5	45%
SD	3	27%
PD	1	9%
**PD** (*n*) In total	1	9%
PD after initial response	0	0%
**Mean time** (*months*) to…		
CR	7.1	
PR	4.0	
Overall response	4.9	
PD	1.0	
**Mean minimum period without known progress** (*months*)	7.6	

## Data Availability

The data presented in this study are available upon request from the corresponding author.
